# Findings and Methodological Shortcomings of Investigations Concerning the Relationship Between Sleep Duration and Blood Pressure: A Comprehensive Narrative Review

**DOI:** 10.3390/jcdd12030095

**Published:** 2025-03-08

**Authors:** Michael H. Smolensky, Ramón C. Hermida, Richard J. Castriotta, Yong-Jian Geng

**Affiliations:** 1 Department of Biomedical Engineering, Cockell School of Engineering, The University of Texas at Austin, Austin, TX 78712, USA; rhermida@uvigo.gal (R.C.H.); yong-jian.geng@uth.tmc.edu (Y.-J.G.); 2 Division of Cardiovascular Medicine, Department of Internal Medicine, McGovern School of Medicine, The University of Texas Health Science Center at Houston, Houston, TX 77030, USA; 3 Bioengineering & Chronobiology Laboratories, Atlantic Research Center for Telecommunication Technologies, Universidade de Vigo, 36310 Vigo, Spain; 4 Bioengineering & Chronobiology Research Group, Galicia Sur Health Research Institute, 36310 Vigo, Spain; 5 Division of Pulmonary, Critical Care, and Sleep Medicine, Department of Medicine, Keck School of Medicine, University of Southern California, Los Angeles, CA 90033, USA; richard.castriotta@med.usc.edu; 6 The Texas Heart Institute at Baylor St. Luke’s Medical Center, Houston, TX 77030, USA

**Keywords:** sleep duration, sleep insufficiency, blood pressure, hypertension, circadian rhythm, investigative methods

## Abstract

Cardiology and sleep societies recommend 7–9 h sleep/night for adults (7–8 h for seniors) and more for youngsters; nonetheless, short sleep duration (SSD) of <7 h/night is epidemic. We searched PubMed for representative investigations, including those cited by meta-analyses, that reported association between SSD and long sleep duration (LSD) of >9 h/night and blood pressure (BP) levels to assess shortcomings of their methods. Studies indicate both SSD and LSD negatively impact BP despite major deficiencies, such as (i) reliance mainly on cross-sectional rather than longitudinal protocols, (ii) inclusion of participants diagnosed with hypertension (HTN) and/or taking antihypertension medications, (iii) assessment of BP and diagnosis of HTN performed by single wake-time office measurement rather than multiple measurements performed by 24 h ambulatory BP monitoring (ABPM), and (iv) determination of SD by subjective recall, single-night polysomnography, or diary recordings rather than objective wrist actigraphy of sufficient duration. The limited number of ABPM-based studies, despite evidencing major shortcomings, particularly (i) assessment for 24 h rather than preferred ≥48 h and (ii) inclusion of subjects diagnosed with HTN and/or taking antihypertension medications, also report association between abnormal SD and elevated 24 h ‘daytime’/wake-time diastolic and systolic (SBP) means plus ‘nighttime’/sleep-time SBP mean and dipping—the latter two indices, in combination, the strongest predictors of major adverse cardiovascular events.

## 1. Introduction

Sleep of adequate duration and quality is imperative for overall well-being and optimal cognitive and physical performance. The National Sleep Foundation, American Academy of Sleep Medicine, and Sleep Research Society recommend 7–9 h sleep/night (n) for adults ≤65 years of age and 7–8 h/n for seniors [1]. The amount of sleep/24 h (nighttime slumber plus daytime napping) recommended for infants and children is even greater—12–16 h for babies 4–11 months of age, 11–14 h for toddlers 1–2 years of age, and 10–13 h for youngsters 3–5 years of age [2]. The amount of sleep/24 h recommended for school children 6–12 years of age and adolescents 13–18 years of age, is, respectively, 9–12 h and 8–10 h [2]. The importance of sleep of sufficient duration and quality for optimal cardiac and vascular health has been acknowledged by the American Heart Association (AHA) through the incorporation in 2022 of sleep duration (SD) into its 8-item Cardiovascular Health Checklist, designated Life’s Essential 8 ™, which additionally includes the variables of nicotine, physical activity, diet, body weight, blood glucose, blood lipids, and blood pressure (BP) [3]. Short sleep duration (SSD) and poor-quality sleep can result in wake-time fatigue, deficient memory consolidation, impaired cognitive performance, compromised concentration, inattentiveness, forgetfulness, mistakes/errors, accidents, and accelerated brain aging [4,5,6,7,8]. They can also degrade mental and physical health [9,10,11,12,13,14] and enhance the risk for hypertension, vascular pathology, and major adverse cardiovascular disease (CVD) events [15,16,17,18,19,20,21,22,23,24,25,26,27,28].

Numerous investigations, whose methods differ widely in design and quality, report association between SSD and/or long SD (LSD) and elevated BP and hypertension (HTN). While various published meta-analyses suggest the magnitude of such associations across investigations [22,23,29,30,31,32], other than assessment of compliance with the guidelines of Preferred Reporting Items for Systematic Reviews and Meta-Analyses (PRISMA) and those Assessing Methodological Quality of Systematic Reviews-2 (AMSTAR-2) [33], none critically evaluated the quality of their investigational methods, i.e., appropriateness of inclusion and exclusion criteria used to constitute study cohorts and the manner in which BP and SD data were gathered. This article aims to (i) narratively summarize the major findings of a large number of relevant investigations that were the basis of past meta-analyses plus others retrieved by search of the PubMed database, utilizing the terms “sleep duration”, “short sleep duration”, and “long sleep duration” paired with “blood pressure” and “hypertension”, and (ii) describe and critique their investigative methods for major deficiencies that likely biased their results. Our intent is not to comprehensively review the findings and methods of all past publications but a large and diverse number of representative ones.

## 2. Sleep Duration Epidemiology

The 2014 US Behavioral Risk Factor Surveillance System survey of 444,306 adults found 34.8% prevalence of unhealthy SSD of <7 h sleep/n, with 11.8% of those surveyed reporting sleep of ≤5 h/n and 23.0% reporting sleep of 6 h/n [34]. An LSD of ≥9 h was reported by 8%. The age-adjusted prevalence of such SSD was greatest among US minority cohorts—non-Hispanic Blacks, American Indians/Alaska Natives, Native Hawaiians/Pacific Islanders, and multiracial non-Hispanic Whites, Hispanics, and Asians. Surveys and databases of countries other than the USA also describe comparable prevalence of SSD (e.g., [35,36,37,38,39]).

Typically, SSD is the consequence of late bedtime. The average bedtime of >1 million American users of the Sleep Cycle App [https://www.sleepcycle.com/sleep-science/what-we-know-about-americas-healthiest-happiest-best-rested/ (accessed on 13 May 2024)] was 23:39 h, and that of 10,000 users of Withings sleep-tracking products was 23:40 h weekdays and 00:10 h weekends [https://blog.withings.com/2014/11/04/study-of-the-sleep-patterns/ (accessed on 13 May 2024)]. A 2017 comprehensive analysis of the world’s most extensive sleep database generated by Fitbit users [https://blog.fitbit.com/sleep-study/ (accessed on 13 May 2024)] found the average bedtime to be 23:36 h, but with generational differences. The bedtime of Baby Boomers (birth year between 1946 and 1964) was earliest, 23:17 h, and was progressively later for Generation Xers (birth year between 1965 and 1980), 23:25 h, Millennials (birth year between 1981 and mid-1996), 23:45 h, and Generation Zers (birth year between 1997 and 2012), 00:08 h. The overall average time of awakening from sleep of all users was 07:17 h, but also with generational differences. It was earliest for Baby Boomers, 06:53 h, and it was progressively later for Generation Xers, 07:00 h, Millennials, 07:28 h, and Generation Zers, 08:12 h. However, the span between bed and rise times is not indicative of total sleep time (TST). Although the average time spent in bed across all Fitbit users was 7 h and 33 min, ~55 min was spent either in restless sleep or wakefulness, such that only 6 h and 38 min in total was spent in actual restful sleep, the TST being somewhat greater for women, 6 h and 50 min, than men, 6 h and 26 min. The mean TST of Baby Boomers was shortest, 6 h and 33 min, and to a slight extent, progressively longer for Generation Xers, 6 h and 34 min, Millennials, 6 h and 40 min, and Generation Zers, 6 h and 57 min. Most concerning is the finding that the average TST across the different generational cohorts was consistently <7–9 h/n, which is less than the amount recommended by the AHA necessary to foster good cardiovascular health. Unfortunately, SSD is not solely characteristic of adults. The 2015 US Youth Risk Behavior Survey revealed a very high prevalence of SSD <9 h on school nights among US children 6–12 years of age and <8 h among adolescents 13–18 years of age [40]. Among pre-teens, the prevalence of SSD was 57.8%, and among adolescent high school students it was even greater, 72.7%. The 2016–2018 US National Survey of Children’s Health not only confirmed the high prevalence of SSD in school-aged children but the very high, 40.3%, prevalence in infants 4–11 months of age [41].

## 3. Sleep Duration, Blood Pressure, and Hypertension

Assessment of BP and diagnosis of HTN in the majority of investigations entailed single time-of-day wake-span measurements obtained by a sphygmomanometer in a clinical setting. A lesser number of them relied on ambulatory BP monitoring (ABPM) devices to repeatedly assess participants at regular intervals both during the wake and sleep spans for a total duration of ≥24 h. Since both the nature of the BP parameters generated by these two methods of assessment and the clinical criteria utilized to make the diagnosis of HTN differ substantially, we present the findings and methods of these two types of investigations in separate sections.

### 3.1. Office Blood Pressure Measurement (OBPM)-Based Sphygmomanometer Investigations

Most investigations relied on the findings of once-a-day wake-time OBPM to assess BP level and determine HTN status of study participants or based on such previous assessment self-report of having been diagnosed with HTN and/or prescribed BP-lowering medication. SD, in most instances, was ascertained by subjective self-report; it was only occasionally assessed objectively by wrist actigraphy. The results of investigations utilizing these methods support the contention that SSD and LSD are associated with risk of elevated systolic (SBP) and/or diastolic BP (DBP) as well as elevated incidence of HTN [31,32,36,37,38,42,43,44,45,46,47,48,49,50,51,52,53,54].

Li and Shang [49] analyzed the data of 12,166 adults free of CVD and diabetes, 30–79 years of age, who participated in one of the four cycles of the cross-sectional US National Health and Nutrition Examination Survey (NHANES) conducted between 2007 and 2014. HTN was defined as having either a wake-time office SBP ≥ 140 mmHg and/or DBP ≥ 90 mmHg, self-report of having HTN, or self-report of taking antihypertensive medication. Habitual SD was ascertained by answering the query “How much sleep do you usually get at night on weekdays or workdays?”, with responses categorized as short (<7 h/n), normal (7–9 h/n), or long (>9 h/n). With 7–9 h/n as the reference, SSD was significantly associated with HTN [odds ratio (OR): 1.20 (1.08–1.33), *p* = 0.001]. In the age- and gender-adjusted model, LSD was also associated with HTN [OR: 1.40 (1.04–1.89), *p* = 0.026)], but not after additionally adjusting for race, marital status, education level, health insurance, body mass index (BMI), physical activity, sedentary time, smoking status, alcohol consumption, and diet [OR: 1.22 (0.89–1.66), *p* = 0.215]. The findings of a follow-up investigation of the same design, utilizing the database of 7,426 participants of the 2015–2016 and 2017–2018 waves of the NHANES, were similar [50]; those who self-reported SSD of <7 h/n evidenced 25% greater incidence of HTN [OR: 1.25 (1.02–1.54), *p* = 0.032] than those who self-reported normal SD of 7–9 h/n.

The cross-sectional study by Gottlieb et al. [45] involved 5,910 participants (49.3% female) 40–100 years of age of the Sleep Heart Health Study (SHHS), a community-based prospective cohort study whose primary objective was to assess the cardiovascular consequences of obstructive sleep apnea (OSA). HTN was defined as wake-time office SBP ≥ 140 mmHg or DBP ≥ 90 mmHg or self-reported use of prescribed antihypertension therapy. Habitual SD was established by replying to the query “How many hours of sleep do you usually get at night or your main sleep period on weekdays or workdays?” The weighted average SD/n of the 7 d week was categorized as <6, 6 to <7, 7 to <8, 8 to <9, and >9 h. The statistically significant (*p* < 0.001) adjusted OR for HTN of those having a SSD of <6 and 6–7 h/n, compared to those having a SD of 7 to <8 h/n, was, respectively, 1.66 (1.35–2.04) and 1.19 (1.02–1.39), and for those having a LSD of 8–9 and ≥9 h/n, respectively, 1.19 (1.04–1.37) and 1.30 (1.04–1.62). This U-shaped relationship between HTN and SD persisted after adjusting for caffeine and alcohol consumption, current smoking habit, insomnia, depression symptoms, sleep efficiency (SE), diabetes mellitus, and previous CVD events.

Pandey et al. [51] examined the data of a cross-sectional investigation of 25,352 North American participants (18% Black), aged 18–85 years of age of the 2009 National Health Interview Survey (NHIS). HTN was defined as positive answer to the query “Have you ever been told by a doctor or other health professional that you have HTN?” Habitual SD was determined through answer to the question “How many hours of sleep do you get on average in a 24 h period?”, with replies rounded up or down to the nearest 1 h. Responses were categorized as short if <7 h, average if 7–8 h, and long if >8 h. Logistic regression analysis revealed that SSD of <6 h/24 h, compared to SD of 6–8 h/24 h, was associated, in both Blacks and Caucasians, with a higher likelihood of reporting diagnosis of HTN [OR: 1.21(1.04–1.41), *p* < 0.001]. Unadjusted logistic regression analysis exploring race/ethnicity interactions indicated Blacks who reported (<6 h/24 h) and long (>8 h/24 h) SD were more likely to have a diagnosis of HTN [respectively, OR: 1.34 (1.02–1.75) and 1.37 (1.07–175), both *p* < 0.01] than those who reported 7–8 h/24 h sleep. LSD of >8 h/24 h of Caucasians was not statistically significantly associated with self-report of having HTN.

Seixas et al. [52] evaluated the data of 261,686 North Americans (17% Black) with a mean age of 48 years partaking in the cross-sectional 2004–2013 NHIS. As in the 2009 NHIS [51], HTN was substantiated as positive reply to the query “Have you ever been told by a doctor or other health professional that you have HTN?” and SD was quantified by reply to the query “How many hours of sleep do you get on average in a 24 h period?”, and again categorized as short if <7 h, average if 7–8 h, and long if >8 h. Some 2.7% of the cohort reported very SSD (<6 h sleep/24 h), 26.82% SSD, 61.59% average SD, and 8.9% LSD. SSD and LSD were more often reported by Blacks, 36.6% and 53.7%, respectively, which translated into a 32% higher prevalence rate (PR) of SSD in Blacks than Caucasians [1.32 (1.29–1.35), *p* < 0.001] and 18% higher PR of reporting LSD [1.18 (1.13–1.23), *p* < 0.001]. Self-reports of having HTN were significantly higher (*p* < 0.001) in those having SSD of <7 h/24 h or LSD of >8 h/24 h, 34.2% and 40.2%, respectively, compared to having average SD of 7–8 h/24 h, 28.6%.

Deng et al. [36] analyzed the database of a cohort of 162,121 non-obese and major disease-free Taiwanese adults (52.6% female), 20–80 years of age, who underwent regularly scheduled medical examinations between 1996 and 2014 conducted by the MJ Health Management Institution of Taiwan. SD was established by response to the question “How many hours do you usually sleep a day (d)?”. Some 18.6% were categorized as short (<6 h/d), 72.8% regular (6–8 h/d), and 8.6% long (>8 h/d) sleepers. Additionally, sleep quality was ascertained by reply to the question “How is your sleep condition in the last month?” with response options being “Difficulty initiating sleep”, “Difficulty maintaining sleep”, “Feeling of non-restorative sleep”, “Use of sleeping pills”, and “Sleep well”. Some 57.6% of the participants were considered to have insomnia based on ≥1 response indicative of such. Individuals were assessed for HTN by wake-time OBPM, utilizing thresholds of ≥140 mmHg for SBP and ≥90 mmHg for DBP. Variables of metabolic syndrome were additionally sampled during follow-up. In analyses fully adjusted for all influential variables (sex, age, BMI, lifestyle, socioeconomic, laboratory analyses, among others), SSD of <6 h/d, compared to regular SD of 6–8 h/d, was significantly (*p* < 0.001) associated with elevated Hazard Ratio (HR) for central obesity by 12% [HR: 1.12 (1.07–1.17)], fasting glucose by 6% [HR: 1.06 (1.03–1.09)], HTN by 8% [HR: 1.08 (1.04–1.13)], low high-density lipoprotein cholesterol by 7% [HR: 1.07 (1.03–1.11)], hypertriglyceridemia by 9% [HR: 1.09 (1.05–1.13)], and metabolic syndrome by 9% [HR: 1.09 (1.05–1.13)]. LSD was associated with reduced risk of hypertriglyceridemia [HR: 0.89 (0.84–0.94)] and metabolic syndrome [HR: 0.93 (0.88–0.99)]. Insomnia symptoms did not modify the effects of SD on these associations.

Fernandez-Mendoza et al. [43] evaluated the relationship between SSD, insomnia, poor sleep, and incident HTN through cross-sectional study of a random sample of 786 adults (51.3% female) categorized as being normotensive (NTN) based on self-report of not using prescribed antihypertension therapy. The participants, who were taking part in a large population-based sleep disorders study among different age groups, were sorted into three mutually exclusive groups of chronic insomnia, poor sleep, and normal sleep. As defined by the authors, chronic insomnia was complaint of insomnia for ≥1 year, poor sleep moderate to severe complaint of difficulty falling asleep, difficulty staying asleep, early final awakening, or non-restorative sleep, and normal sleep absence of complaints of both insomnia and poor sleep. Participants underwent single-night polysomnography (PSG) in a sleep laboratory in which BP was measured in the supine position ~2 h prior to the initiation of PSG. Normal BP status was defined as SBP < 120 mmHg and DBP < 80 mmHg, pre-hypertension (pre-HTN) status as SBP ≥ 120 and <140 mmHg and/or DBP ≥ 80 and <90 mmHg, and HTN status as SBP ≥ 140 mmHg and/or DBP ≥ 90 mmHg. SD was determined by the single-night PSG, with SSD defined as sleep <6 h and LSD as sleep ≥6 h. Compared to those who slept ≥6 h, adjusting for sex, race, age, caffeine intake, tobacco use, alcohol consumption, depressive mood, sleep-disordered breathing, diabetes mellitus, obesity, and baseline BP, highest incidence of incident HTN was displayed by SSD insomniacs [OR: 3.75 (1.58–8.95), *p* = 0.012)]. The OR for incident HTN in SSD poor sleepers, compared to normal quality sleepers of SD ≥ 6 h, was also high [1.8 (1.04–3.12), *p* = 0.036], but it became statistically insignificant when controlling for obesity [1.62 (0.92–2.83), *p* = 0.09]. Finally, the incidence of incident HTN was not significantly increased in those who complained of chronic insomnia or poor sleep and slept ≥6 h the night of PSG.

Vgontzas et al. [53] explored the combined impact of chronic insomnia and poor sleep and objectively assessed SD on the prevalence of HTN through a cross-sectional population-based study of 1,741 subjects (54.4% female) who on average were 48.7 of age (sd ± 13.5). The original purpose of the investigation was to determine the age distribution of sleep-disordered breathing. Subjects were classified as having (i) insomnia if experiencing for ≥1 year difficulty falling asleep, remaining asleep, and/or early final awakening; (ii) poor sleep if experiencing moderate to severe difficulty falling asleep, remaining asleep, early final awakening, and unrefreshing sleep; or (iii) normal sleep if not experiencing any of the signs and symptoms of insomnia and poor sleep. SD was ascertained by single-night laboratory PSG and categorized as ≤5 h, 5–6 h, and ≥6 h. BP was measured once while in the supine position in the evening ~2 h prior to PSG. HTN was defined as SBP >140 mmHg, DBP > 90 mmHg, or self-report of taking antihypertensive medication. The OR for HTN of those who complained of insomnia and had a SSD of ≤5 h the night of PSG, compared to participants who did not complain of insomnia and had a SD of >6 h the night of PSG, was 5.12 [(2.2–11.8), *p* < 0.01]. The OR for HTN of those with complaint of insomnia and SSD of 5–6 h the night of PSG, compared to those without complaint of insomnia or poor sleep and SD of >6 h the night of PSG, was 3.53 [(1.6–7.9), *p* < 0.01], and the OR for HTN of the coupled effect of mild insomnia and SSD of ≤5 h sleep the night of PSG was 2.43 [(1.4–4.3), *p* < 0.01]. Finally, the OR for HTN of those who complained of insomnia or poor sleep and had SD >6 h sleep the night of PSG was increased but not significantly (NS), 1.31 [(0.6–2.5), NS] and 0.79 [(0.5–1.2), NS], respectively.

Javaheri et al. [48] analyzed the data of 238 illness-free adolescents (48.3% female) 13–18 years of age participating in the Cleveland Children’s Sleep and Health Study (CCSHS). They underwent 5–7 d of wrist actigraphy to objectively quantify, under usual conditions of life, their sleep efficiency (SE), calculated as total time spent asleep/total time spent in bed × 100. Additionally, they underwent single-night PSG. BP was reported as the average of three repeated measurements made at 21:00 h just before PSG, at 08:00 h just after PSG when subjects were in supine position, and at 09:30 h following PSG when subjects were in seated position. The prevalence of pre-HTN, i.e., SBP and/or DBP ≥ 90th percentile according to age, gender, and height; low SE of ≤85%; and SSD of ≤6.5 h/n, respectively, was 14%, 26%, and 11%. The OR of pre-HTN for those having a lower-than-normal SE, compared to those having normal SE, determined by analyses adjusted for sex, BMI, and socioeconomic status was 3.5 [(1.5–8.0), *p* = 0.003]. The OR for pre-HTN was only marginally insignificant, 2.54 [(0.93–6.9), *p* = 0.068], in those having SSD of ≤6.5 h/n, compared to those having SD of >6.5 h/n, when adjusted for the above-stated influential variables. The adjusted analyses additionally revealed adolescents having low SE ≤ 85% exhibited an average 4 mmHg higher SBP than adolescents having normal SE of >85%.

Gangwisch et al. [44] through a longitudinal study design explored the association between SD and development of HTN in a cohort of 4,810 NTN individuals 32–86 years of age who participated in the 1982–1984, 1986, 1987, and 1992 follow-up waves of the first US NHANES. Persons were excluded if, by wake-time OBPM, SBP was >140 mmHg, DBP > 90 mmHg, or if self-reporting having a diagnosis of HTN at or before the first (1982–1984) wave of the NHANES. Habitual SD was ascertained from answer to the question of the 1982–1984 survey “How many hours of sleep do you usually get a night or when you usually sleep?”, and development of HTN was substantiated during the subsequent 8–10 years of follow-up, at the times of the 1986, 1987, and 1992 follow-up waves of the NHANES, by self-report of having received a diagnosis during a physician or hospital visit or it being listed on one’s death certificate. In unadjusted analyses, participants of the entire sample who self-reported sleeping ≤5 h/n, compared to 7–8 h/n, were more likely to have been diagnosed with HTN during follow-up [HR: 1.76 (1.37–2.56), *p* < 0.001]. However, the findings differed by age. Participants 32–59 years of age when recruited who self-reported sleeping ≤5 h/n, compared to 7–8 h/n, were more likely [HR: 2.10 (1.58 to 2.79), *p* < 0.001] to have been diagnosed with HTN during follow-up, while subjects 60–86 years of age who self-reported sleeping ≥9 h/n, compared to 7–8 h/n, were more likely [HR: 1.54 (1.03–2.30), *p* < 0.001] to have been diagnosed with HTN during follow-up.

Kim et al. [38] conducted a longitudinal 2.4-year follow-up study to examine the risk of developing HTN according to the trajectory of one’s SD over time. A cohort of 106,385 healthy young and middle-aged Korean women and men, mean age of 40.6 years, without previous medical history of HTN, CVD, or sleep disorders, were followed. Participants were enrolled in the Kangbuk Samsung Health Study, an ongoing prospective cohort study of Korean adults who partake in annual or biennial comprehensive medical examinations. SD was obtained by an item of the Pittsburgh Sleep Quality Index that queried the actual amount of sleep obtained per typical 24 h period the previous month, with responders categorized as short (≤6 h/24 h), moderate (7 h/24 h), and long (≥8/24 h) sleepers. HTN was defined as wake-time office measured SBP ≥140 mmHg or DBP ≥90 mmHg or self-report of current use of antihypertensive medication. Criteria for inclusion of data of subjects for analyses were complete data file and attendance of ≥3 follow-up clinical visits (visit 1: baseline, visit 2: exposure period, visit 3: outcome, i.e., NTH or HTN). Changes in SD between the first and the second clinical visits constituted the on average 1.3-year exposure period to one’s persistent or altered, shorter or longer, SD. During the exposure span, the SD of 42.3% of the female and 50.7% of the male subjects remained stable. A decrease of ≥1 h of sleep/24 h was observed in 32% of the female and 27% of the male subjects, while an increase of ≥1 h of sleep/24 h was observed in 25.7% of female and 22.4% of male subjects. Moreover, during the exposure span, ~50% of the subjects reported ≤5 h, 38.2% ≤6 h, and 2.7% ≥8 h sleep/24 h. During the mean study span of 2.4 years, 4,750 incident cases of HTN were documented. Decrease of SD by ≥1 h/24 h and persistent SSD of <6 h sleep/24 h were both associated with risk of incident HTN (at least *p* = 0.01). However, gender differences were identified. In women incident HTN was associated with shorting of SD, while in men incident HTN was associated with lengthening of SD. Women for whom SSD of ≤6 h/24 h persisted, compared with women for whom SD of 7 h/24 h persisted, were more likely to have developed HTN during follow-up [HR: 1.34 (1.05–1.70)], while men for whom SD increased from ≤6 h to ≥8 h/24 h were likely to have developed HTN during follow-up [HR: 1.32 (1.01–1.73)]. Further, the shortening or lengthening of SD by ≥2 h/24 h, compared with no alteration of SD, during follow-up was statistically significantly (at least *p* = 0.01) associated with incident HTN in both women [HR: 1.46 (1.08–1.98)] and men [HR: 1.31 (1.10–1.56)].

Huang et al. [37] followed 3,178 Chinese NTN adults (57% female), 30 to >75 years of age, who participated in the 5-year follow-up of the 2004 and 2009 China Health and Nutrition Surveys. SD was self-reported in answer to the query “How many hours do you usually sleep each day, including daytime and nighttime in the last year?” Answers were grouped into three categories: ≤7, 8–9, and ≥10 h sleep/d. Trajectories of SD during follow-up were categorized either as persistent/unchanged or altered. HTN was defined by a positive reply to the query “Has a doctor ever told you that you suffer from high blood pressure?” Subjects who did not respond or who stated “no” or “unknown” were assessed by mercury sphygmomanometer utilizing diagnostic HTN thresholds of ≥140 mmHg for SBP and ≥90 mmHg for DBP. The prevalence of persistent short (≤7 h/d), normal (8–9 h/d), and long (>9 h/d) SD during follow-up was, respectively, 9.1%, 37.7%, and 2.3%. Both at baseline and follow-up, prevalence of HTN was significantly (*p* < 0.001) higher in those having SD of ≤7 h/d and ≥10 h/d than those having SD of 8–9 h/d. Moreover, the prevalence of incident HTN was higher in participants having persistent SSD (31.5%), persistent LSD (40.3%), or altered SD of ≥1 h/d (29.1%), compared to those having and maintaining normal SD (21.1%). Overall, participants who during follow-up persistently slept ≤7 h/d or ≥10 h/d or whose SD changed by ≥1 h/d, compared with those who persistently slept 8–9 h/d, showed significantly (always *p* < 0.001) higher risk ratio of incident HTN, 1.38 (1.12–1.69), 1.56 (1.17–2.07), and 1.3 (1.14–1.49), respectively.

### 3.2. Ambulatory Blood Pressure Monitoring (ABPM)-Based Investigations

A lesser number of ABPM-based than OBPM-based studies have explored the impact of SD on BP in cohorts of middle-aged, older [55,56,57,58,59,60], and young [61] adults and adolescents [62,63]. Unfortunately, the results of many of these investigations are confounded because the duration of BP assessment was too short (≤24 h), BP monitoring was conducted as an inpatient rather than outpatient procedure, and pre-classification of participants as either NTN or HTN was accomplished not by 24 h ABPM and associated BP thresholds but by single wake-time OBPM and associated BP thresholds. Moreover, the findings of some investigations that examined the relationship between SD and the major variables of ‘daytime’/wake-time, ‘nighttime’/sleep-time, 24 h BP means, and ‘nighttime’/sleep-time BP dipping phenotype are muddled by the effects of antihypertension medications taken by participants.

The cross-sectional study by Kim et al. [57] evaluated the impact of SD and other factors on ‘nighttime’ BP and dipping phenotype of 221 NTN and 359 untreated and 401 treated HTN Koreans (47.1% female; average age of 53.9 years) enrolled in the Korean multicenter nationwide prospective registry of ambulatory BP monitoring (KORABP) trial. BP status of each participant was categorized as HTN if taking antihypertensive medication, SBP ≥ 140 mmHg and/or DBP ≥ 90 mmHg by wake-time OBPM, or elevated ABPM-determined 24 h mean of SBP ≥ 130 mmHg and/or DBP ≥ 80 mmHg. SD was calculated as the difference between diary-recorded times of the commencement and conclusion of sleep during the single night of 24 h ABPM. Sleep quality was categorized by answer to the query “How was the sleep last night?” according to a Likert scoring scale of ‘hardly slept’ (1 point), ‘poor sleep’ (2 points), ‘not so bad sleep’ (3 points), and ‘very good sleep’ (4 points). BP was measured every 15 or 30 min during the ‘daytime’ (authors’ terminology) and every 30 or 60 min during the ‘nighttime’. ABPM profiles were considered suitable for analysis if they contained ≥20 ‘daytime’ and ≥7 ‘nighttime’ valid BP values after removal of erroneous ones according to the following criteria: (i) SBP < 7 0 mmHg or >250 mmHg, (ii) DBP < 40 mmHg or >150 mmHg, and (iii) pulse pressure < 20 mmHg or >150 mmHg. ‘Daytime’ BP was defined as the average of all of valid measurements obtained between 08:00 and 21:00 h. ‘Nighttime’ BP was defined as the average of all valid measurements obtained during actual sleep. Additionally, nighttime’ BP determined by the authors’ so-termed narrow fixed-interval method was defined as the average of all of the valid BP values obtained between 00:00 and 05:00 h, and ‘nighttime’ BP during deep sleep was defined as the average of all of valid BP values obtained between 2 h after sleep onset and 1 h before sleep awakening. The 24 h average BP was calculated as ‘nighttime’ BP × actual SD/24 + ‘daytime’ BP × (24 − actual SD)/24. Extent of SBP decline during ‘nighttime’ sleep from its awake ‘daytime’ level was categorized as extreme dipping if ≥20%; normal dipping if ≥10% and <20%; non-dipping if between 0% and <10%; and reverse dipping if <0%. The mean SD the night of ABPM among participants was 7.2 h. Self-ratings of sleep quality per the Likert scoring scale of 1 to 4 were, respectively, 15.5%, 30.0%, 30.4%, and 24.2%. Among those with HTN, extreme dipper, dipper, non-dipper, and reverse dipper phenotypes were characteristic, respectively, of 58 (7.63%), 277 (36.45%), 325 (42.76%), and 100 (13.16%) participants. Multiple linear regression analyses of the data of participants classified as having HTN revealed statistically significant association between ‘nighttime’ SBP and SD (*p* < 0.001), and also ‘nighttime’ SBP and sleep quality (*p* < 0.001). Statistically significant association was additionally substantiated between extent of ‘nighttime’ SBP dipping and sleep quality (*p* < 0.001), but not SD (*p* = 0.08). SBP dipping was positively associated with sleep quality [OR: 1.16 (1.03–1.30)], while SBP reverse dipping was negatively associated with sleep quality [OR: 0.73 (0.62–0.86)]. However, interpretation of the findings of this study is confounded by several factors, particularly absence of information on habitual SD as opposed to reliance only on the SD during the single-night of 24 h ABPM, unknown aggressiveness, in terms of number and dose of prescribed BP-lowering medications, of the clinical management of elevated BP, and also circadian timing of such medications —upon awakening from sleep vs. before bedtime for sleep—that exerts differential effect on wake-time and sleep-time BP and consequently extent of sleep-time SBP dipping [64,65,66,67] of the >50% of the total sample of treated HTN study subjects.

Shulman et al. [60] performed post hoc cross-sectional analysis of data derived from the baseline 24 h ABPM profiles obtained from two cohorts of adult participants who had not taken antihypertensive medications for ≥2 months. One cohort was composed of 66 participants (50% female) of the Lifestyle Modification in Blood Pressure Lowering Study (LIMBS), and the other was composed of 153 participants (13.7% female) of the Penn Icelandic Sleep Apnea Study (PISAS). The stated goal of this investigation was to examine the association between SD and BP parameters provided by 24 h ABPM. LIMBS was initially designed as a randomized, parallel group, non-blinded, prospective, controlled trial to evaluate the effects of yoga and BP education on development of HTN. Inclusion criteria were adults 18–75 years of age who, by wake-time OBPM, evidenced SBP between 130 and 160 mmHg. Exclusion criteria were BMI >40 kg/m^2^, kidney disease, diabetes mellitus, CVD, smoking, extensive alcohol consumption, and, in women, pregnancy. PISAS was initially designed as an observational study to elucidate the clinical and molecular characteristics associated with BP change in adults 40–65 years of age without medical history of treatment for OSA. Subjects were excluded if evidencing an unstable or new medical condition ≤1 month prior to screening, severe and uncontrolled arterial HTN (SBP > 180 mmHg/ DBP > 110 mmHg), BMI > 40 kg/m^2^, routine consumption of >2 alcoholic beverages/d, excessive caffeine use (>10 cups/d), and, in women, pregnancy. In LIMBS, BP was measured every 20 min during ‘daytime’ (authors’ terminology) and every 30 min during ‘nighttime’. An ABPM profile was deemed valid if between 06:00 and 00:00 h it contained ≥28 (≥51.9%) of the 54 programmed measurements and between 00:00 and 06:00 h it contained ≥6 (≥50%) of the 12 programmed measurements. In PISAS, BP was sampled every 30 min throughout the 24 h. An ABPM profile was considered valid if between 06:00 and 00:00 h it contained ≥16 (≥44.4%) of the 36 programmed measurements and between 00:00 and 06:00 h it contained ≥6 (≥50%) of the 12 programmed measurements. However, the authors failed to state if and by what methods the BP 24 h profiles were edited for erroneous values. Clock time of sleep onset and offset plus SD were determined by the difference in study diaries of listings of bed and awakening times made by participants during the single 24 h ABPM. In LIMBS, the 24 h mean SBP was significantly (*p* < 0.001) higher by 12.7 mmHg in those having SSD of <7 h/n (mean SD 5.4 h) compared to those having longer SD of ≥7 h/n (mean SD 8.6 h/n). Moreover, difference between both the mean wake-time SBP, 148.1 vs. 136.0 mmHg (*p* < 0.001), respectively, and mean sleep-time SBP, 133.4 vs. 124.1 mmHg (*p* = 0.029), respectively, was greater among shorter than longer sleepers. There was no association between SD and extent of nocturnal BP dipping; moreover, prevalence of asleep BP dipping did not differ significantly (*p* = 0.205) between those having SSD of <7 h/n sleep (65% prevalence) vs. longer SD of >7 h/n sleep (47% prevalence). Finally, shorter, compared to longer, SD was more often linked with uncontrolled or sustained HTN (47% vs. 20%, *p* = 0.033). In PISAS, the 24 h mean SBP was significantly (*p* > 0.005) higher by 4.7 mmHg in those having SSD of <7 h/n (mean SD 5.8 h/n) than those having longer SD of ≥7 h/n (mean SD 8.3 h/n). However, ‘daytime’ SBP (134.6 vs. 131.2 mmHg, *p* = 0.053) and ‘nocturnal’ SBP (118.0 vs. 114.8 mmHg, *p* = 0.098) did not differ significantly between those who had a shorter than longer SD and neither did the prevalence of sleep-time BP dipping, 65% vs. 65% (*p* = 0.97).

Doyle et al. [55] studied 300 adults (50% female), 21–70 years of age, enrolled in the North Texas Heart Study, a longitudinal investigation of stress and atherosclerotic risk. Inclusion criteria were age >21 years, residence in Denton County, Texas, and written and verbal fluency in the English language. Exclusion criteria were engagement in shift work, cognitive impairment; prior myocardial infarction; cardiac interventions, such as coronary artery bypass surgery and implanted cardiac defibrillator, and in women pregnancy the past year or anticipating such during the study period. SD and SE were determined objectively by 48 h wrist actigraphy. BP was assessed by an atypical ABPM protocol—‘daytime’ and ‘nighttime’ (authors’ terminology) ABPM sessions not conducted during the same 24 h span. Measurements were considered artefactual or erroneous and discarded if (i) SBP < 70 mmHg or >250 mmHg, (ii) DBP < 45 mmHg or >150 mmHg, or (iii) SBP/DBP ratio < [1.065 + (0.00125 × DBP)] or >3.0. ‘Daytime’ SBP and DBP means were derived from the average of all respective valid values obtained by ABPM during the two separate assessments that extended from 07:00 to 22:00 h. ‘Nighttime’ SBP and DBP means were derived mainly utilizing valid values obtained between 00:00 and 05:00 h of the single night of ABPM when wrist actigraphy confirmed ≥90% of that time was spent in sleep. If ≥90% of this time period was not composed of sleep, but a significant sleep period that overlapped it was, a sleep window of ~5 h duration was identified, and its corresponding BP values were averaged to represent sleep BP. ABPM was programmed to sample BP at random times during individual 45 min intervals of the ‘daytime’ and ‘nighttime’ periods. Among the 300 study participants, 48 (16%) lacked information on mean SE and SD, 4 (1%) lacked mean ‘daytime’ SBP and DBP values, and 58 (19%) lacked mean ‘nighttime’ SBP and DBP values. The average SBP and DBP were, respectively, 146.15 and 84.31 mmHg over the two ‘daytime’ ABPM sessions. The wrist actigraphy-determined mean SD and SE of subjects the night of ABPM were, respectively, 6.7 h and 82.52%. A lower than average SD was associated with higher mean ‘daytime’ SBP (*p* < 0.05) but not DBP (*p* > 0.21). Analyses that focused on data from the single night of BP revealed that a lower than average SD was significantly (*p* < 0.001) associated with higher mean ‘nighttime’ SBP and DBP. Moreover, lower SE was associated with significantly (*p* < 0.001) higher ‘daytime’ SBP, but not higher DBP, and higher ‘nighttime’ SBP and DBP. When both SD and SE were analyzed together, lower SE was associated with higher ‘daytime’ SBP, while shorter SD was associated with higher ‘nighttime’ SBP and DBP. However, the findings of this investigation are confounded by its unique ABPM sampling paradigm and also its unique definition of the nighttime sleep period.

Friedman et al. [56], utilizing a cross-sectional design, investigated the relationship between self-reported SD and characteristics of the SBP and DBP profile obtained by 24 h ABPM. The sample consisted of 525 patients (62.5% female) referred to the Mount Sinai Hospital Hypertension Clinic between 1 July 2005 and 30 June 2008 for ABPM and whose ABPM profiles were complete for ≥70% of the programmed measurements. The authors did not additionally specify the minimum proportion of the programmed measurement per wake and sleep span of each 24 h profile required to qualify data of each subject for inclusion in statistical analyses. BP was measured every 20 min during the wake period and every 30 min during the sleep period; however, no particulars were provided if and how the ABPM-derived data were edited for potentially erroneous values. ‘Nocturnal’ (authors’ terminology) BP decline was defined as percent decline in SBP overnight relative to ‘daytime’ SBP, calculated as 100 × (1 − ‘nighttime’ SBP/’daytime’ SBP), with non-dipping defined as a <10% ‘nocturnal’ fall of SBP. ’Day–night’ SBP difference was calculated as ‘nighttime’ SBP minus the ‘daytime’ SBP. ‘Morning’ SBP surge was calculated as mean SBP during the 2 h following awakening minus mean SBP during the 1 h of sleep that included lowest SBP, and elevated ‘morning’ SBP surge was defined as ≥18.0 mmHg. ‘Nocturnal’ HTN was distinguished as ‘nighttime’ SBP ≥ 125 or ‘nighttime’ DBP ≥ 75 mmHg. SD and daytime wake and nighttime sleep spans were derived from diary-recorded clock times of retiring to sleep at night and awakening in the morning during the single span of 24 h ABPM. Participants, classified as NTN or HTN based upon wake-time OBPM and self-reported use of prescription BP-lowering medications, were divided into one of four groups. One group was composed of 108 NTN persons who did not take BP-lowering medication and whose SBP was <135 mmHg and DBP < 85 mmHg. The three other groups (417 participants in total) were composed of HTN subjects according to wake-time OBPM whose (i) SBP and/or DBP were/was controlled by medication—SBP < 135 mmHg and DBP < 85 mmHg; (ii) SBP and/or DBP were/was uncontrolled by medication—SBP ≥ 135 mmHg and/or DBP ≥ 85 mmHg; and (iii) SBP and/or DBP were/was elevated but untreated. Some 18.5% of the participants slept ≤5 h and 7.6% ≥9 h when undergoing ABPM. Both SBP non-dipping and morning SBP surge of subjects of all four study groups were affected by SD. Across the four groups of subjects, the OR for SBP non-dipping per 1 h decrease in SD was 1.12 [(1.00–1.24); *p* = 0.04], while that for elevated morning SBP surge per 1 h increase in SD was 1.13 [(1.02–126); *p* = 0.02]. Overall, sleep deficit in the form of SSD, based upon the single night of 24 h ABPM, was associated with SBP non-dipping and attenuated morning SBP surge, while sleep surfeit in the form of LSD was also associated with SBP non-dipping and increased morning SBP surge. However, the findings of this study are confounded by the potential misclassification of BP status of participants due to potential masked HTN and NTN by to the reliance on wake-time OBPM for their classification, plus for two of the four study groups the unknown impact of dose strength and circadian timing of the antihypertension medications on all of the ABPM-derived outcome variables.

Fujikawa et al. [61] assessed the association between SD and BP through study of 331 “healthy” medical students (39% female) 21–33 years of age. BP was sampled every 30 min by ABPM throughout a single 24 h span. However, there was no stated information if and how the BP profiles were edited for potentially erroneous BP values, and nor was there stated information concerning the minimum proportion of the respective wake and sleep-time measurements required to qualify the individual BP profiles for inclusion in statistical analyses. HTN was defined according to the 2007 guidelines of the European Society of Hypertension/European Cardiology College, i.e., mean 24 h SBP > 125 mmHg and/or DBP > 80 mmHg. SD was calculated as the difference between the clock times recorded in one’s study diary of the commencement and termination of slumber, which was supplemented with information on body position and physical activity acquired by sensors of the ABPM device. SSD was defined as a sleep length of ≤5 h the night when ABPM was performed. Some 67 (20.2%) of the medical students exhibited an elevated SBP and/or DBP 24 h mean indicative of HTN, with the incidence being higher in male (30.7%) than female (3.9%) medical students. The overall 24 h mean BP, calculated as one-third of all valid SBP values + two-thirds of all valid DBP values, was significantly negatively correlated with SD (r −0.207, *p* < 0.001), with DBP being significantly (*p* = 0.03) greater in the 51 SSD (≤5 h) study participants (31.4% of sample) than the 280 (18.2%) non-SSD (≥5 h) study participants. Unfortunately, the authors did not report association between SD and the clinically meaningful ABPM-derived parameters of sleep-time SBP mean and SBP dipping, which are more sensitive prognosticators of CVD risk than the overall 24 h BP or 24 h SBP and DBP means that are of low clinical utility [64,65,66,67].

Mezick et al. [62] investigated associations between SE and ambulatory BP in a cohort of 244 Black (56.5%) and Caucasian (43.5%) high school students (53.3% female) between 14 and 19 (mean 15.7) years of age whose data profiles were protocol correct for all study variables. None of the students had a medical history of CVD or kidney disease, and none took sleep, cardiovascular, or psychiatric medications. SD and SE were primarily determined by 7 d wrist actigraphy, with activity counts summed per 1 min interval. BP was assessed by 48 h ABPM, with sampling performed every 30 min between 07:00 and 22:00 h and every 60 min between 22:00 and 07:00 h. SBP and DBP readings that differed by ±3 sd from one’s respective mean values were excluded as outliers; however, other important criteria for certifying 24 h ABPM profiles suitable for statistical analyses, were not specified. The calculation of the average ‘nighttime’ (authors’ terminology) BP was based on ≥5 valid measurements obtained during the participant’s self-reported sleep span. Outcome variables were as follows: 24 h BP, defined as the average of all valid measurements obtained throughout 48 h ABPM; ‘nighttime’ (authors’ terminology) BP, defined as the average of all valid BP readings obtained during the sleep span, designated as 22:00–07:00 h; ‘daytime’ BP, defined as the average of all valid BP readings during the waking span, designated as 07:00–22:00 h; and sleep–wake BP ratio, defined as the ratio of the average ‘nighttime’ BP divided by the average ‘daytime’ BP. The average SD/n across the 7 n of wrist actigraphy was 6.45 h (range 4.27–9.22 h), and the average SE/n was 82.7% (range 67.8–94.1%). Average decline among subjects of ‘nighttime’ SBP and DBP was, respectively, ~13% and ~20%. After adjustment for age, sex, and BMI, Black, compared to Caucasian, adolescents evidenced significantly (*p* < 0.05) higher 24 h DBP, ‘nighttime’ SBP, and ‘daytime-nighttime’ SBP ratio. Shorter SD among all adolescents, independent of race, was associated with significantly higher 24 h SBP and DBP, largely driven by higher ‘nighttime’ SBP and DBP over the range of average sleep length/n between ≤9.2 and ≤5.8 h. Shorter SD was also associated, but not in a statistically significant manner, in the same direction with ‘daytime’ SBP and DBP. Moreover, adolescents, independent of race, who evidenced shorter, rather than normal, SD were more likely to be classified as pre-HTN, exhibit SBP >120 and/or DBP >80 mmHg [OR: 0.66 (0.46–0.97)]; have elevated ‘daytime’ SBP >130 Hg or DBP >80 mmHg [OR: 0.65 (0.42–0.98)]; and be non-dippers—<10% decline in ‘nocturnal’ SBP or DBP [OR: 0.66 (0.44–0.99)]. Follow-up analyses revealed associations between SD and BP tended to be more prevalent in Caucasian than Black adolescents.

Meininger et al. [63] studied an ethnically diverse cohort of 366 pre-adolescents and adolescents (54% female) 11–16 years of age attending public schools. They were assessed by ABPM, with BP measurements made every 30 min throughout the 24 h span of a school day and night. SD was appraised by wrist actigraphy, with activity counts summed per 1 min interval. An ABPM study was considered valid if ≥85% of all the programmed BP measurements were present. However, no information was provided regarding if and how individual BP profiles were edited for erroneous readings, and nor was information provided regarding the minimal number of valid daytime and nighttime values required for individual BP profiles to be considered protocol correct for data inclusion into statistical analyses. Based on one’s BMI, 21% of the adolescents were obese and 18% overweight, and per wake-time OBPM 10% of the adolescents were classified as pre-HTN and 4% as HTN. The mean SD/n of the entire cohort was 6.83 h (sd ± 1.36 h), and when including daytime napping the total sleep/d was 7.23 h (±1.67 h). The average nighttime SD of girls was longer (6.98 h) than boys (6.66 h), and that of Hispanic adolescents (7.02 h) was shorter than non-Hispanic White (7.13 h) and other ethic adolescents (7.26 h), but it was shortest in non-Hispanic Blacks (6.39 h). Controlling for daytime nap duration and demographic factors, anthropometric indices, daytime physical activity, body position and location at the time of BP measurements, each additional 1 h of nighttime sleep was associated with 0.36 mmHg lower ‘daytime’ (authors’ terminology) SBP (*p* = 0.02). Controlling for nighttime SD and all co-variables, each additional 1 h of ‘daytime’ sleep was significantly associated with 1 mmHg lower ‘daytime’ SBP (*p* < 0.001). Only ‘nighttime’ SD was significantly (*p* = 0.001) associated with lower ‘nighttime’ SBP (−0.69 mmHg per additional 1 h sleep). Further, ‘daytime’ sleep was (*p* < 0.001) associated with lower ‘daytime’ DBP (−0.84 mmHg per additional 1 h sleep), whereas ‘nighttime’ SD was not significantly related to ‘daytime’, ‘nighttime’, or 24 h DBP.

## 4. Discussion

The goals of this narrative review are to (i) summarize the major findings relating to the association between SD and BP level and HTN status, and (ii) critique the nature and quality of the methods of reported investigations, among others, that comprised past meta-analyses [22,23,29,31,32,68,69,70]. Detailed critique of the diversity of such investigative protocols has not been previously performed and thus constitutes a novel attribute of our article.

Past meta-analyses, such as the ones conducted by Itani et al. [22], Jike et al. [23], Hosseini et al. [29], Wang et al. [31,32], Dai et al. [68], Li et al. [69], and Nurrobi et al. [70], plus many recent prospective investigations indicate both SSD and LSD, in comparison to normal SD, are associated with increased risk of elevated BP and HTN. The exact mechanisms of these associations are not yet well understood. Some propose SSD acts as a stressor that resets sympathovagal balance causing enhanced activation of the sympathetic nervous system and hypothalamic–pituitary–adrenal axis. Accordingly, this results in increased circulation of adrenaline, noradrenaline, rennin, angiotensin, aldosterone, and cortisol, inducing blood vessel constriction that results in elevated BP [71,72,73]. The mechanisms of the LSD-associated elevation of BP are different. LSD is often the consequence of compromised chronic mental or physical health status. This includes sleep apnea, type 2 diabetes, coronary heart disease, respiratory system disorders, and pain syndromes, like osteoarthritis that is exacerbated nocturnally by TNFα, IL-1β, and other somnogenic immunological modulators [23,25,74,75,76,77,78]. Thus, the prevalence of elevated BP and HTN associated with LSD is seemingly mediated by these and other medical conditions. Nonetheless, despite overwhelming evidence of the link between BP level/HTN and SD, critical review of the methods of reported investigations reveals major deficiencies that seemingly confound discovery of the true strength of their association. These include how (i) BP was measured and HTN was diagnosed; (ii) how SD was assessed, quantified, and classified; (iii) how inclusion and exclusion criteria were established for recruitment of study participants; and (iv) how investigations were designed and conducted.

### 4.1. Deficiencies of the Method of Blood Pressure Assessment and Diagnosis of Hypertension

Both SBP and DBP are circadian rhythmic in healthy sleepers. In diurnally active persons, they rise from reduced levels ~1 h before morning awakening, increase progressively during daytime wakefulness, but with brief minor midday dip, to peak in the late afternoon/early evening, and decrease thereafter during sleep to lowest values, with the day time peak-to-nighttime trough difference in SBP and DBP typically amounting to, respectively, 10–20 and 5–10 mmHg [79]. This 24 h pattern is orchestrated by a multitude of circadian processes [80], including those of circulating blood volume and body temperature, which declines just before sleep consequent to the increased dissipation of body heat to the external environment through redistribution of as much as 50% of the cardiac output from the central systemic circulation to the peripheral arteriovenous anastomoses of the glabrous skin, must notably that of the palms of the hands and soles of the feet [67,81,82]. Despite knowledge of the circadian variation in BP and its potential impact on the accurate assessment and classification of subjects, determination of SBP and DBP level and differential diagnosis of HTN vs. NTN in the vast majority of past studies were accomplished not by ABPM, which enables complete assessment and parameterization of 24 h BP pattern, but by wake-time OBPM, which is representative of BP level only a unique single time of the 24 h. Moreover, in various investigations, classification of BP status relied on self-report of having previously received a diagnosis of HTN and/or taking prescription antihypertension medications that, too, were based on findings of wake-time OBPM. Even though OBPM is the most popular means of measuring of BP level and diagnosing HTN, it has at least four inherent limitations that potentially confound determination of the true strength of the relationship not only between SD and SBP and DBP level but incident HTN.

First, given the predictable-in-time variation of ~20% in SBP and ~10% in DBP during the 24 h, values derived from single wake-time OBPM are unlikely to be representative of those at other times, particularly during sleep [79]. Wake-time OBPM is particularly likely to be non-representative of BP level and/or status of elderly persons and those who have chronic renal disease, type 2 diabetes, and sleep disorders like OSA. This is because their BP 24 h pattern is commonly characterized by an abnormally elevated sleep time, non-dipping SBP, and/or DBP, which cannot be detected or quantified by wake-time OBPM [83,84,85,86,87].

Second, the clinical environment in which measurements are performed typically elicits a pressor reaction, termed ‘white coat effect’, which results in acute elevation of BP. When substantial, it can result in an invalid diagnosis of HTN, termed ‘white coat HTN’, ‘isolated clinical HTN’, or more properly ‘masked NTN’, because in settings other than the clinical one, BP is normal. Additionally, a diagnosis of NTN made by wake-time OBPM may be invalid because of BP being abnormally elevated in settings outside the clinical one during this time of the day; this is termed ‘masked HTN’. The combined prevalence of masked NTN and masked HTN is >35%. Additionally, >20% of adults diagnosed as NTN by wake-time OBPM evidence sleep-time HTN in the form of an abnormal non-dipping or rising BP phenotype when assessed by around-the-clock ABPM. Thus, collectively, sole dependency on wake-time OBPM may result in potential misclassification of as many as 50% of evaluated individuals [88,89].

Third, interpretation of wake-time OBPMs for diagnostic purposes relies on BP thresholds recommended by medical guidelines that are periodically revised [90]. Thresholds recommended for SBP and DBP in 2003 by the 7th Report of the Joint National Committee on Prevention, Detection, Evaluation, and Treatment of High Blood Pressure [91] were, respectively, 140 and 90 mmHg, while those recommended in 2017 by the American College of Cardiology/American Heart Association [92] were, respectively, 130 and 80 mmHg. Adoption in the US of these thresholds resulted in 14% increase in the prevalence of HTN [93].

Fourth, previously conducted outcomes trials and meta-analyses suggest the definition and manner of making the diagnosis of HTN require reconsideration since they document major adverse CVD events are significantly better predicted by the ABPM-derived asleep SBP mean than wake-time OBPM [89,94,95,96,97,98,99,100,101,102,103]. For example, the meta-analysis by Roush et al. [103], although reporting the wake-time office SBP and ambulatory awake and asleep SBP means significantly predict CVD risk when each is entered separately into analyses, established the sleep-time SBP mean to be the *sole* independent predictor of risk when all of BP variables were entered into analyses simultaneously. Additionally, many trials and meta-analyses substantiate CVD risk is also forecasted by attenuated sleep-time SBP decline [89,99,101,102,104,105]. Thus, elevated asleep SBP mean and blunted sleep-time SBP decline jointly constitute the most sensitive predictors of CVD risk, independent of wake-time OBPM or ABPM-determined wake-time or 24 h SBP or DBP means [103,106]. Accordingly, such findings support the proposition asleep SBP mean and extent of SBP dipping, in combination, constitute the true definition of HTN [99,100,101,102] and, therefore, should be the basis for its diagnosis and thus classification of participants of SD investigations through proper application of cost-effective around-the-clock ABPM [107,108].

### 4.2. Deficiencies of ABPM-Based Investigations

Results of the relatively small number of investigations reviewed herein entailing ABPM are also confounded because of non-adherence to recommended guidelines for its proper application [88,101]. In all but one investigation involving adolescents [62], the duration of ABPM was limited solely to 24 h, rather than desired ≥48 h, resulting in unacceptably high error in estimating derivable BP parameters [107]. Conducting ABPM throughout a continuous span of at least 48 h is necessary to accurately estimate the true wake-time, sleep-time, and 24 h SBP and DBP means, as well as accurately calculate the extent of sleep-time SBP and DBP dipping. Furthermore, when ABPM is performed for ≥48 h, measurements can be made less often, every 30 or even 60 min, rather than every 15 or 20 min, intervals to improve one’s tolerance to assessment both during the wake and sleep period without compromising accuracy of estimating BP parameters [107]. Additionally, reported 24 h SBP and DBP means of most ABPM-based investigations were likely biased because of the methods used to calculate them. Their derivation was prejudiced by the greater number of measurements that over-represent the longer ≥16 h wake span when particularly in young healthy persons BP tends to be the most elevated compared to the shorter ≤8 h sleep span when BP tends to be lowest during the 24 h period. Moreover, reliance on the 24 h SBP and DBP means is clinically less meaningful than reliance on the asleep SBP mean and amount of SBP dipping, because the former are not sensitive indicators of true HTN or CVD risk [89,99,101,102,104,105]. Furthermore, the methods section of most ABPM-based publications [56,60,61,63] failed to state if and how derived BP profiles were edited for artefactual and erroneous values. Also, some [55,61,62] failed to provide particulars of the overall minimum number of valid BP readings per 24 h span, and even a lesser number provided such per wake and sleep span [57,60,62], required to qualify BP profiles as protocol correct for inclusion into statistical analyses. Too, the diverse manner in which BP findings were reported—as biologically irrelevant designated ‘daytime’ and ‘nighttime’ means as opposed to biologically relevant designated wake-time and sleep-time means—in the different ABPM-based investigations not only compromises accurate quantification of the relationship between SD and assessed BP variables [55,88,109,110] but confounds comparison of findings between investigations. Finally, the protocol used to conduct ABPM was sometimes improper. For example, in the study by Doyle et al. [55], ABPM measurements were made at random times per individual 45 min interval during two consecutive 07:00–22:00 h spans to determine the ‘daytime’ SBP and DBP means, and, during a separate single 22:00–07:00 h nighttime span, ABPM measurements were again made in this manner to determine the asleep SBP and DBP means, mainly utilizing data obtained between 00:00 and 05:00 h.

### 4.3. Deficiencies of the Quantification of Sleep Duration and Definition of Short and Long Sleep Duration

The manner of ascertaining SD was inconsistent among investigations. Most of them relied on subjective recall to answer the query of “How many hours of sleep do you usually get a night or when you usually sleep” [44], “How much sleep do you usually get at night on weekdays or workdays?” [49,50], “How many hours of sleep do you usually get at night or your main sleep period weekdays or workdays” [45], “How many hours of sleep do you get on average in a 24 h period?” [51], “How many hours of sleep do you get on average?” [52], “How many hours do you usually sleep a day?” [36], “How many hours do you usually sleep each day, including daytime and nighttime in the last year?” [37], or an item of the Pittsburgh Sleep Quality Index [38]. The subjective report of SD is problematic because of potential recall bias [111,112]; moreover, it can be significantly underestimated in those with insomnia [113]. A couple of investigations utilized the SD of a single night of laboratory PSG [43,53] or a single night of 24 h ABPM [56,57,61,63] to classify participants, thus raising the question of whether the findings of these reports represent only an acute transient situational relationship rather than a true chronic relationship between SD and BP. In this regard, the assessment of one’s sleep architecture by polysomnography and SD may be compromised by the so-called “first night effect” [114,115]. Only a few investigations quantified SD objectively by wrist actigraphy for just 2 [55] or as many as 5–7 d [48,62]. Finally, definition of SSD varied between investigations. It was defined in some as <7 h/n or <7 h/ 24 h [34,37,45,49,50,51,52,60], while in others it was <6.5 h [48], <6 h [36,38,43], or <5 h [61] per night or 24 h. Definition of LSD also varied between investigations. It was defined in some investigations as >6 h/n [43,53], in several others as >8 h/n or >8 h/ 24 h [36,38,45,51], and in a few others as 9 h/n [49,50] or >10 h/d [37].

### 4.4. Deficiencies in the Design and Conduct of Investigations

The majority of past studies were cross-sectional in design. Only a limited number of them were the preferred longitudinal ones that differed in duration from ~2 [38], 5 [37] or 8–10 [44] years.

### 4.5. Deficiencies of the Source of Blood Pressure and Sleep Duration Data

Several investigations entailed analyses of existing databases of national surveys, such as the NHANES [44,49,50], NHIS [51,52], Behavioral Risk Factor Surveillance System survey [34], US National Survey of Children’s Health [41], Youth Risk Behavior Survey [40], Sleep Heart Study [45], Cleveland Children’s Health Study [48], or Kangbuk Samsung Health Study [38]. Others entailed analyses of data from investigations originally designed for a different primary purpose, such as LIMBS and PISAS [60], North Texas Heart Study [55], Penn State Cohort study [43], KORABP [57], China Health and Nutrition Survey [37], and Taiwanese MJ Health Database study [36]. 

### 4.6. Deficiencies and Disparities of Inclusion and Exclusion Criteria of Study Participants

The nature of the study cohorts varied considerably among investigations. The composition of most of them were mainly middle-aged or elderly adults; only a small number of them involved adolescents or young adults [40,41,48,61,62,63]. Some 24 h ABPM-based studies concerned groups of untreated and treated HTN participants, and in the case of the latter, BP either was or was not controlled by antihypertension medication [20,55,56]. One study involved individuals who were untreated for OSA but without specification of its prevalence in the cohort [60], thereby confounding accurate determination of the specific impact of SD vs. OSA on the 24 h ‘daytime’/awake-time, ‘nighttime’/sleep-time SBP and DBP means plus SBP dipping phenotype. Additionally, studies that entailed persons taking prescription antihypertension therapy surely impacted SBP and DBP values assessed by OBPM and wake-time and sleep-time SBP and DBP means plus dipping status derived by ABPM. Moreover, the time when BP medication was taken by such study participants was not reported. Numerous trials document the effect of antihypertension medications on the wake-time and sleep-time SBP and DBP means and extent of their dipping can differ substantially when ingested in the evening/at bedtime compared to upon awaking/morning [64,65,66,67].

## 5. Perspectives: SSD in the Context of the American Heart Association’s Cardiovascular Health Life’s Essential 8 ^TM^

SSD is highly prevalent in adults, as substantiated by published reports cited herein and analyses of databases of wearable devices [https://blog.withings.com/2014/11/04/study-of-the-sleep-patterns/; https://blog.fitbit.com/sleepstudy/; https://www.sleepcycle.com/sleep-science/what-we-know-about-americas-healthiest-happiest-best-rested/ (all accessed on 13 May 2024)], as well as children and adolescents [40,41,116] likely promoted by late-night use of electronic devices that fosters or reinforces evening chronotype [117,118]. The 2006 Sleep in America Poll [http://sleepfoundation.org/sites/default/files/2006_summary_of_findings.pdf (accessed on 10 June 2024) reported ~97% of adolescents possesses ≥1 electronic device—television, computer, mobile phone, or video game console—in their bedroom, and the 2010 Pew Research Center survey [https://www.pewresearch.org/internet/2010/04/20/chapter-three-attitudes-towards-cell-phones/2010 (accessed on 10 June 2024)] reported 84% of adolescents at least occasionally utilized a mobile phone in their bedroom at night to search the internet, play games, and socialize with peers—activities that induce psychological arousal and result in delayed sleep onset, sleep difficulty, and sleep insufficiency that translates into SSD and risk of daytime fatigue [119,120,121,122,123,124,125,126].

SSD is linked with various behaviors and habits that negatively impact physical activity, blood glucose, blood lipids, BP, smoking, BMI, and diet—variables that comprise the American Heart Association’s Cardiovascular Health Life’s Essential 8 ^TM^ [3]. For example, SSD is associated with abnormal eating patterns, particularly delayed dinner time and late-night snacking of unhealthy foods high in calories, carbohydrates, and saturated fat [127,128,129]. Maintenance of healthy body weight and BMI and preservation of metabolic health depend not only on total number of calories consumed in excess of energy expenditure per 24 h but also circadian time when most of them are consumed. Weight gain by diurnally active children and young adults is enhanced if foods are habitually ingested toward the end of the activity span as a late dinner meal and/or as late-night snacks, which has been associated with breakfast skimming or skipping [130,131,132,133,134,135,136,137,138,139,140,141,142]. In this regard, the 2021 and 2022 Food and Health Surveys of the International Food Information Council [https://foodinsight.org/wp-content/uploads/2021/05/IFIC-2021-Food-and-Health-Survey.May-2021-1.pdf; https://foodinsight.org/survey-spotlight-snacking (both accessed on 10 June 2014)] reported young persons, relative to seniors, were 10-fold more likely to consume savory/salty foods and/or candy, chocolate, cookies, cake, and ice cream in the evening and late at night after 23:00 h.

Regular late-night consumption of snacks rich in carbohydrates and saturated fats in the milieu of late bedtime and SSD promotes not only weight gain but metabolic syndrome and vascular atherosclerotic pathology [128,129,143,144,145,146,147,148,149,150,151,152]. Metabolic syndrome, defined according to the updated National Cholesterol Education Program Adult Treatment Panel III [153], is the manifestation of ≥3 of the following five features: waist circumference > 40 inches for males and >35 inches for females, serum triglycerides ≥ 150 mg/dL, serum low density lipoprotein cholesterol concentration < 40 mg/dL for men and <50 mg/dL for women, fasting glucose ≥ 100 mg/dL, and SBP ≥ 130 mmHg or DBP ≥ 85 mmHg. Risk of these conditions is additionally fostered by sedentariness [154,155,156] through the essentially addictive use of electronic devices. Furthermore, habitual sedentariness, late-night eating, and snacking behaviors promoted by late bedtime-associated SSD foster risk of OSA, whose hypopneas and apneas cause cardiac stress plus risk of sleep-time HTN and non-dipping 24 h SBP patterning, the latter two BP features being the strongest prognosticators of future adverse CVD events [85,99,100,106,157,158]. Finally, the lifestyle habit of SD due to late bedtime, particularly prominent in adolescents and young adults, promotes and reinforces late chronotype—preference for late bedtime and late morning awakening time—that is associated with unhealthy lifestyle and habits, including poor diet, tobacco use, and alcohol consumption [125,159,160]. Thus, SSD that results from late bedtime, particularly in adolescents and young adults, is concerning not only because of its linkage to elevated SBP and DBP and incident HTN but because of its linkage to unhealthy behaviors and habits that jeopardize metabolic and vascular health [16,18,19,22,26,27,28,36] that lead to atherosclerotic vascular pathology [15,17,20,21,24] and heightened risk of major adverse CVD events [161,162].

Although there is clear evidence for the association between HTN with SSD, and to much lesser extent LSD, there is urgent need for future well-designed prospective longitudinal, but also cross-sectional, investigations devoid of methodological shortcomings of past investigations, as specified above, that incorporate ≥48 h ABPM and long-term wrist actigraphy to accurately assess the true strength of relationships.

## Data Availability

No new data were created in this review. Data sharing is not applicable to this article.
